# A Comparison of Low-Complexity Real-Time Feature Extraction for Neuromorphic Speech Recognition

**DOI:** 10.3389/fnins.2018.00160

**Published:** 2018-03-28

**Authors:** Jyotibdha Acharya, Aakash Patil, Xiaoya Li, Yi Chen, Shih-Chii Liu, Arindam Basu

**Affiliations:** ^1^HealthTech NTU, Interdisciplinary Graduate School, Nanyang Technological University, Singapore, Singapore; ^2^School of Electrical and Electronic Engineering, Nanyang Technological University, Singapore, Singapore; ^3^Institute of Neuroinformatics, University of Zurich and ETH Zurich, Zurich, Switzerland

**Keywords:** silicon cochlea, neural engineering framework, extreme learning machine, neuromorphic, real-time

## Abstract

This paper presents a real-time, low-complexity neuromorphic speech recognition system using a spiking silicon cochlea, a feature extraction module and a population encoding method based Neural Engineering Framework (NEF)/Extreme Learning Machine (ELM) classifier IC. Several feature extraction methods with varying memory and computational complexity are presented along with their corresponding classification accuracies. On the N-TIDIGITS18 dataset, we show that a fixed bin size based feature extraction method that votes across both time and spike count features can achieve an accuracy of 95% in software similar to previously report methods that use fixed number of bins per sample while using ~3× less energy and ~25× less memory for feature extraction (~1.5× less overall). Hardware measurements for the same topology show a slightly reduced accuracy of 94% that can be attributed to the extra correlations in hardware random weights. The hardware accuracy can be increased by further increasing the number of hidden nodes in ELM at the cost of memory and energy.

## 1. Introduction

Considerable progress has been made recently in machine learning for speech recognition tasks with the developments in traditional Gaussian Mixture Models and Hidden Markov Models to the more recent deep neural networks (Hinton et al., 2012). However, these models require very complicated processing of the input speech and are not suited for simple sensor nodes with limited power; nor do they perform well in the presence of large background noise (cocktail party problem). In contrast, the human auditory system is able to perform sound stream segregation easily. This has led to an interest in studying the biological auditory system and developing silicon models of cochleas that operate in an event-driven asynchronous fashion (Liu et al., 2014) much like the neurons in the auditory pathway. These event-based asynchronous cochlea sensors implement a bio-mimetic filtering circuit that produces spikes at the output in response to input sounds (Chan et al., 2007; Liu and Delbruck, 2010; Liu et al., 2014). The AEREAR2 sensor has been used previously for typical speech recognition problems such as speaker identification (Chakrabartty and Liu, 2010; Li et al., 2012) and digit recognition (Abdollahi and Liu, 2011; Anumula et al., 2018). The inter-spike intervals and channel specific spike counts are used as features for these tasks. High classification accuracy (95%) was reported using these features for a speaker independent digit recognition task using a software implementation of support vector machine (SVM) based implementation (Abdollahi and Liu, 2011). However, this method required the storage of the entire spike response of the cochlea channels to one spoken digit so that the spikes can be pre-processed prior to classification resulting in huge memory requirements.

In parallel, there has been considerable progress in developing neural models of cognition and a particularly popular one based on population coding is the Neural Engineering Framework (NEF) (Eliasmith and Anderson, 2004; Eliasmith et al., 2012). proposes a framework for neural simulations where the input is non-linearly encoded using random projections and linearly decoded to model the required function. The typical NEF architecture consists of three layers, the input layer, a hidden layer consisting of a large number of non-linear neurons and an output layer consisting of linear neurons. In the encoding phase, the inputs are multiplied with random weights and passed to the non-linear neurons. The non-linear function can be any neural model from the spiking Leaky-Integrate-and-Fire model to more complex biological models (Stewart, 2012). With the use of recurrent connections, NEF can also be used for modeling even dynamic functions. NEF has been proved to be an efficient tool for implementing large scale brain models like SPAUN (Stewart et al., 2012) and therefore, is being widely used in neuromorphic research community.

A similar model has been separately developed in the machine learning community. Termed as the Extreme Learning Machine (ELM) (Huang et al., 2006), it also uses a three layered architecture with random projection of the input and linear decoding. It is essentially a feedforward network and does not have feedback connections allowed in NEF—hence, it may be considered as a sub-category of NEF architectures. It has been used in a variety of applications ranging from neural decoding (Chen et al., 2016) and epileptic seizure detection (Song et al., 2012) to speech recognition (Deng et al., 2017) and big data applications (Akusok et al., 2015) in the past. Low power hardware implementations of this algorithm have also been reported recently (Yao and Basu, 2017). Since we also use a feedforward network in this work, we will refer to our algorithm as ELM in the rest of the paper acknowledging that it can be referred to as NEF as well.

In this work, we bring together these two developments of neuromorphic spiking cochlea sensors and population encoding based ELM hardware to lay the groundwork for a low power bio-inspired real-time sound recognition system. Several different low-complexity feature extraction methods that do not require storage of entire spike trains are explored in this paper and tradeoffs between memory/computation requirements and recognition accuracy are presented. Measured accuracy results using the silicon cochlea in Liu et al. (2014) and ELM chip in Yao and Basu (2017) are presented for the TIDIGITS dataset with 11 spoken digit classes. Though the entire processing of the signal does not use spike times, our method still uses “physical” computation in the cochlea and NEF/ELM blocks which is the essence of neuromorphic engineering as described in Mead (1990).

The remainder of this paper is organized as follows: section 2 details the hardware and the proposed methods. section 3 computes the hardware complexity for the proposed methods. section 4 reports the results for both software simulation and hardware measurements and finally, section 5 presents a discussion on the obtained results.

## 2. Materials and methods

**Figure 1:**
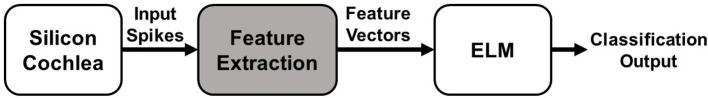
Block diagram of the proposed speech recognition system. The shaded block for feature extraction is implemented in software in this work while the other two blocks are implemented in hardware.

The basic architecture of our proposed speech recognition system is shown in Figure 1. The speech input is acquired by the Dynamic Audio Sensor and the spikes produced are then passed to the feature extraction block. The extracted features are then sent to an Extreme Learning Machine for classification. For the experiments in this paper, we have simulated the feature extraction block in software only, but the feature extraction techniques described here can easily be implemented in hardware using standard microcontrollers. Measured results from hardware are presented for the cochlea and the ELM chip.

### 2.1. Silicon cochlea and recordings

**Figure 2:**
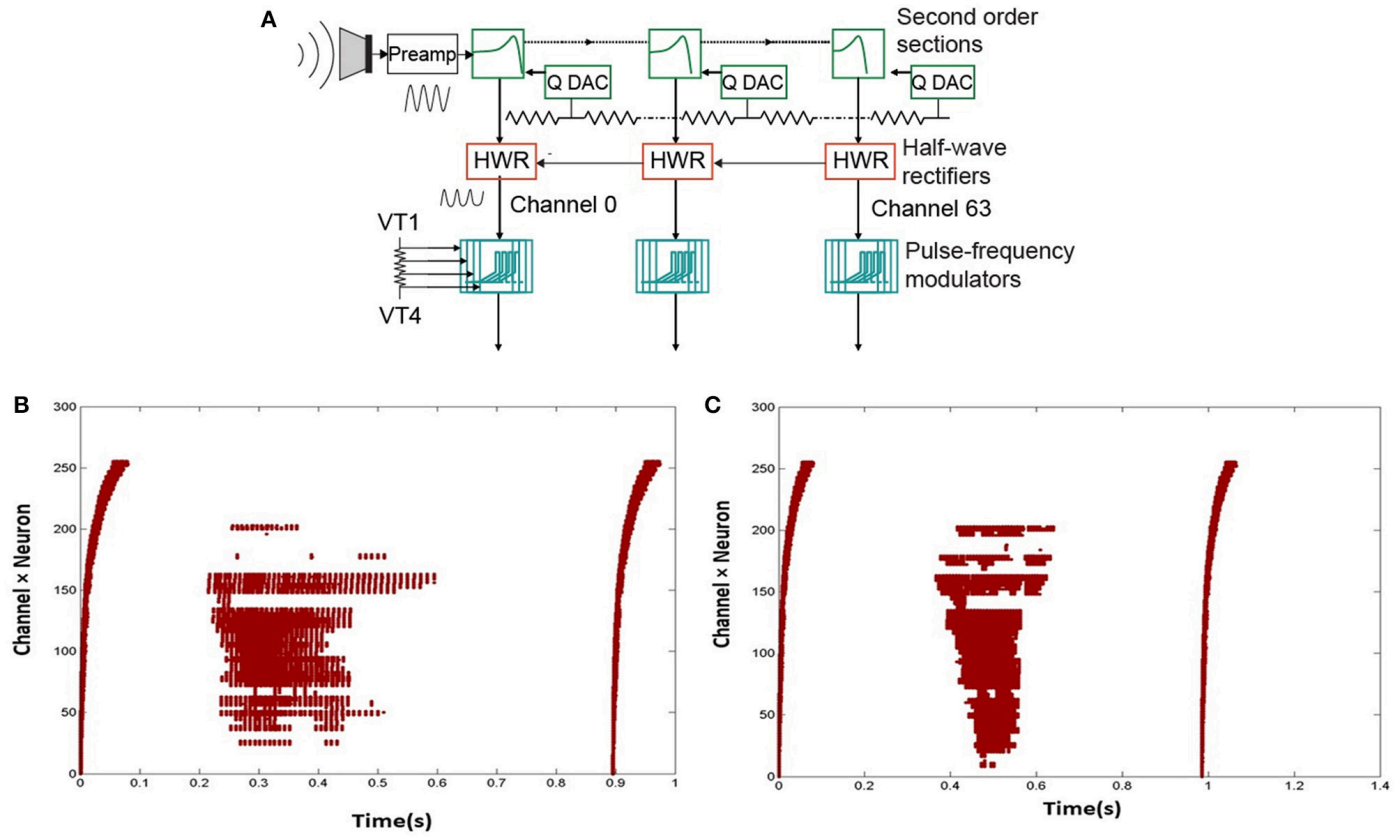
**(A)** Circuit architecture of one ear of the Dynamic Audio Sensor (adapted from Liu et al., 2014). The input goes through a cascaded set of 64 bandpass filters. The output of each of the filters is rectified. This rectified signal then drives an integrate-and-fire neuron model. **(B,C)** Two sample spikes of digit “2.” Dots correspond to spike outputs from the 64 channels of one ear of the cochlea.

The N-TIDIGITS18 dataset (Anumula et al., 2018) used in this work, consists of recorded spike responses of a binaural 64-channel silicon cochlea (Chan et al., 2007) in response to audio waveforms from the original TIDIGITS dataset (Leonard, 1984). The silicon cochlea and later generations of this design, model the basilar membrane, inner hair cells and spiral ganglion cells of the biological cochlea. The basilar membrane is implemented by a cascaded set of 64 second-order band-pass filters, each with its own characteristic frequency. The output of each filter goes to an inner hair cell block which performs a half-wave rectification of its input. The output of the inner hair cell goes to a ganglion cell block implemented by a spiking neuron circuit. The spike output is transmitted off-chip using the asynchronous address-event representation (AER). The binaural chip is connected to microphones emulating left and right ears. The circuit architecture of one ear is shown in Figure 2A. Circuit details are described in Chan et al. (2007) and Liu et al. (2014).

In the recordings, impulses are added at the beginning and end of the audio digit files so that the start and end points of the spike recordings are visible. The impulses lead to spike responses from all channels. Figures 2B,C show two sample spikes of digit “2”. Dots correspond to spike outputs from the 64 channels of one ear of the cochlea.

### 2.2. Preprocessing methods

To obtain the feature vectors from the spike recordings of the silicon cochlea, we used the spike count per window or bin for two modes of binning with two binning strategies which resulted in four preprocessing techniques as shown in Table 1. In the methods described, we used bins of width W and used counters to count the number of spikes across different channels within that bin. The output of the *i*th bin can be represented as *X*_*W*_(*i*) where *X*_*W*_ is a [1 × *C*] vector containing spike counts across *C* channels. Next, we cascaded the bin outputs to produce the feature vectors. The 4 modes differ in the choice of *W* and the number of vectors to be cascaded.

**Table 1:** Preprocessing methods.

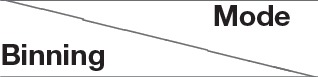	**Time**	**Spike count**
Fixed Bin Size	1A	2A
Fixed No. of Bins	1B	2B

#### 2.2.1. Binning modes

We used two modes for binning the cochlea images to extract features. The first one is time based binning (1A, 1B) where the whole spike sample is divided into several bins based on time or duration of the sample (*T*_*sample*_). The second one is spike count based binning (2A, 2B) where we binned the spike trains based on the total number of spikes in the sample (*N*_*sample*_). While the time based strategy captures the spike density variation in cochlear images quite well, it completely ignores the temporal variation (longer vs. shorter samples). On the other hand, the spike count based strategy captures the temporal variation but ignores the spike density variation (dense vs. sparse samples).

#### 2.2.2. Binning strategies

For all modes, we used two binning methods, (A) fixed bin size and (B) fixed number of bins. These methods are described below for the time based binning mode only to avoid repetition. A similar philosophy applies to the case of spike count based binning.

##### 2.2.2.1. Fixed number of bins

**Figure 3:**
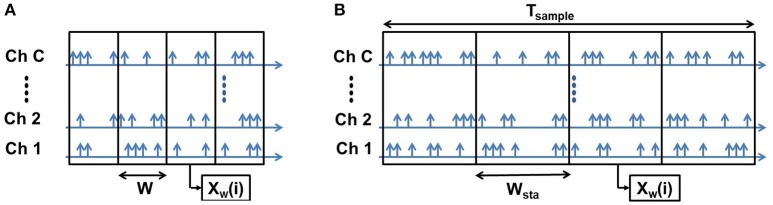
Fixed number of bins: Both the short **(A)** and long **(B)** samples have the same number of bins but the bin width (W) is shorter for short samples and longer for long samples.

In this method, the total number of bins per sample is fixed or static. As a result, in the time mode of binning, the longer samples produce longer bins than shorter samples (as shown in Figure 3). If the number of bins per sample is fixed at *B*_*sta*_, and the corresponding bin width is *w*_*sta*_ for a sample, the total duration of the sample, *T*_*sample*_ is given by:

(2.2.1) $$\begin{array}{r} T_{sample}=w_{sta}\times B_{sta} \end{array}$$

In this method, we explicitly set the value of *B*_*sta*_ and *w*_*sta*_ is determined by:

(2.2.2) $$\begin{array}{r} w_{sta}=T_{sample}/B_{sta}. \end{array}$$

If the total number of spikes per sample is denoted as *N*_*sample*_ and the average number of spikes/bin/channel is denoted by ${\bar{n}}_{spikes}$, we can write:

(2.2.3) $$\begin{array}{r} N_{sample}=B_{sta}\times C\times {\bar{n}}_{spikes} \end{array}$$

The output of each bin (*Xw*(*i*)) is cascaded to produce the feature vector *F* = [Xw(1) Xw(2)…Xw(B)]. So the dimension of the feature vector is *C* × *B*_*sta*_. Thus, there is a clear trade-off between the feature vector size and temporal resolution of the bins. Higher temporal resolution leads to a larger feature vector size and therefore higher classification complexity and vice-versa. The primary disadvantage of this method is that it requires a priori information about the duration of total spike count of the sample before the binning. So, the entire sample needs to be stored first and afterwards binning is done on the sample. Thus, the memory requirement of this method is quite high and the latency is equal to the sample duration. Finally, use of a dynamic bin size removes inter-sample variability of temporal resolution by performing an intrinsic normalization. The longer samples are compressed as a result of longer bin sizes while shorter samples expanded as a result of shorter bin sizes. This is the feature extraction method used in previous work such as Abdollahi and Liu (2011).

In the spike count mode, the total number of spikes *N*_*sample*_ summed across all channels and time is divided into a fixed number of bins (*B*_*sta*_) leading to a limit (*N*_*sample*_/*B*_*sta*_) on total number of spikes per bin. Whenever this limit is reached, it defines the formation of a bin. Spike counts in all channels are frozen to create a feature vector and this process repeats.

##### 2.2.2.2. Fixed bin size

**Figure 4:**
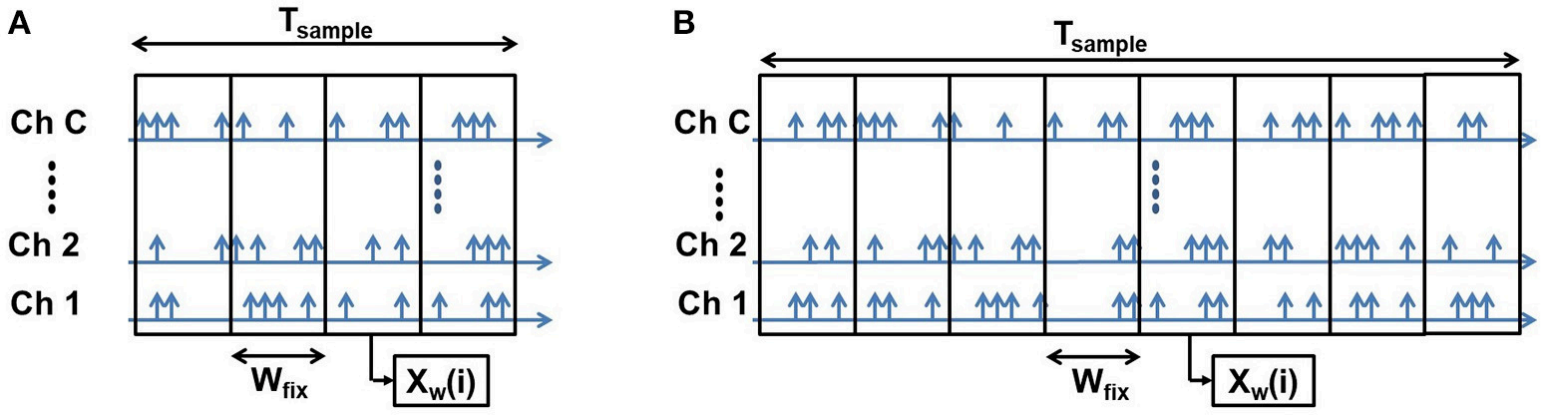
Fixed Bin Size: Both the short **(A)** and long **(B)** samples have the same bin width (W). A short sample produces smaller number of bins and a long sample produces larger number of bins.

In the fixed bin size method, the size of bins is predetermined in terms of time duration or spike count based on the mode of binning. As a result, the longer samples produce larger number of bins while shorter samples produce smaller number of bins (as shown in Figure 4).

Denoting the number of bins per sample using this strategy as *B*_*fix*_, setting the bin width to *w*_*fix*_ and using the same notations as the previous method, we can write:

(2.2.4) $$\begin{array}{r} T_{sample}=w_{fix}\times B_{fix} \end{array}$$

In this method, we explicitly set the value of *w*_*fix*_ and the corresponding value of *B*_*fix*_ is determined by:

(2.2.5) $$\begin{array}{r} B_{fix}=T_{sample}/w_{fix} \end{array}$$

The total number of spikes per sample is given by:

(2.2.6) $$\begin{array}{r} N_{sample}=B_{fix}\times C\times {\bar{n}}_{spikes} \end{array}$$

As the number of bins produced by the samples (*B*_*fix*_) is different for different samples and the ELM classification algorithm requires a fixed feature vector size, we needed to find an optimum number of bins that produce overall high accuracy irrespective of sample duration. Larger number of bins results in increased feature vector size which in turn makes the classification task more difficult and computationally expensive while smaller number of bins result in feature vectors that sample the spike recordings coarsely and thus, miss the finer variations over the sample durations. Our initial experiments suggested that, for number of bins 8-12 the classification accuracy is optimum. Therefore, we decided to fix the number of bins to 10. So, The dimension of the feature vector is 10 × *C*. Based on the bin size and total sample duration, one of two cases can occur:

**Case I:** *B*_*fix*_ ≥ 10

If the sample produced more than 10 bins, we will keep the output of only first 10 bins to produce the feature vectors while ignoring the rest. These bins are then cascaded to produce the feature vector *F* = [*Xw*(1)*Xw*(2)…*Xw*(10)]. In this case,

(2.2.7) $$\begin{array}{r} T_{sample}\geq w_{fix}\times 10 \end{array}$$

For this case, we only use a fraction of total spikes to produce the feature vector. If the number of spikes used is given by *N*_*used*_, we can write:

(2.2.8) $$\begin{array}{r} N_{used}=10\times C\times {\bar{n}}_{spikes}\leq B_{fix}\times C\times {\bar{n}}_{spikes}=N_{sample} \end{array}$$

**Case II:** *B*_*fix*_ < 10

For the samples that produce less than 10 bins for a given bin size, zero padding is used to produce the feature vectors. In this case,

$$\begin{array}{l} T_{sample}<w_{fix}\times 10 \end{array}$$

For this case, we use all the spikes in the sample to produce the feature vector. So,

(2.2.9) $$\begin{array}{r} N_{used}=B_{fix}\times C\times {\bar{n}}_{spikes}=N_{sample} \end{array}$$

So, generalizing the two cases, we can express *N*_*used*_ as:

(2.2.10) $$\begin{array}{r} N_{used}=min\left\{10\times C\times {\bar{n}}_{spikes},B_{fix}\times C\times {\bar{n}}_{spikes}\right\} \end{array}$$

There is no need to store the sample in memory for this method since the feature vectors are directly produced from the samples with predetermined bin sizes. Thus, memory required for this method is quite low. As we require only 10 bin outputs to form a feature vector, the latency is independent of the sample duration unlike the previous strategy. The primary drawback of this strategy is that to obtain fixed feature vector sizes, we have to use a fixed number of bins (10 in our case) to produce the feature vectors and therefore, for larger samples, the rest of the bin outputs are discarded. So, there is a loss of information in this strategy. Moreover, as the bin size is fixed, this method does not provide any input duration normalization like the earlier strategy. A similar fixed spike count based frame size strategy has been used by Moeys et al. (2016) for feature extraction.

### 2.3. Classification methods

#### 2.3.1. Extreme learning machine: algorithm

**Figure 5:**
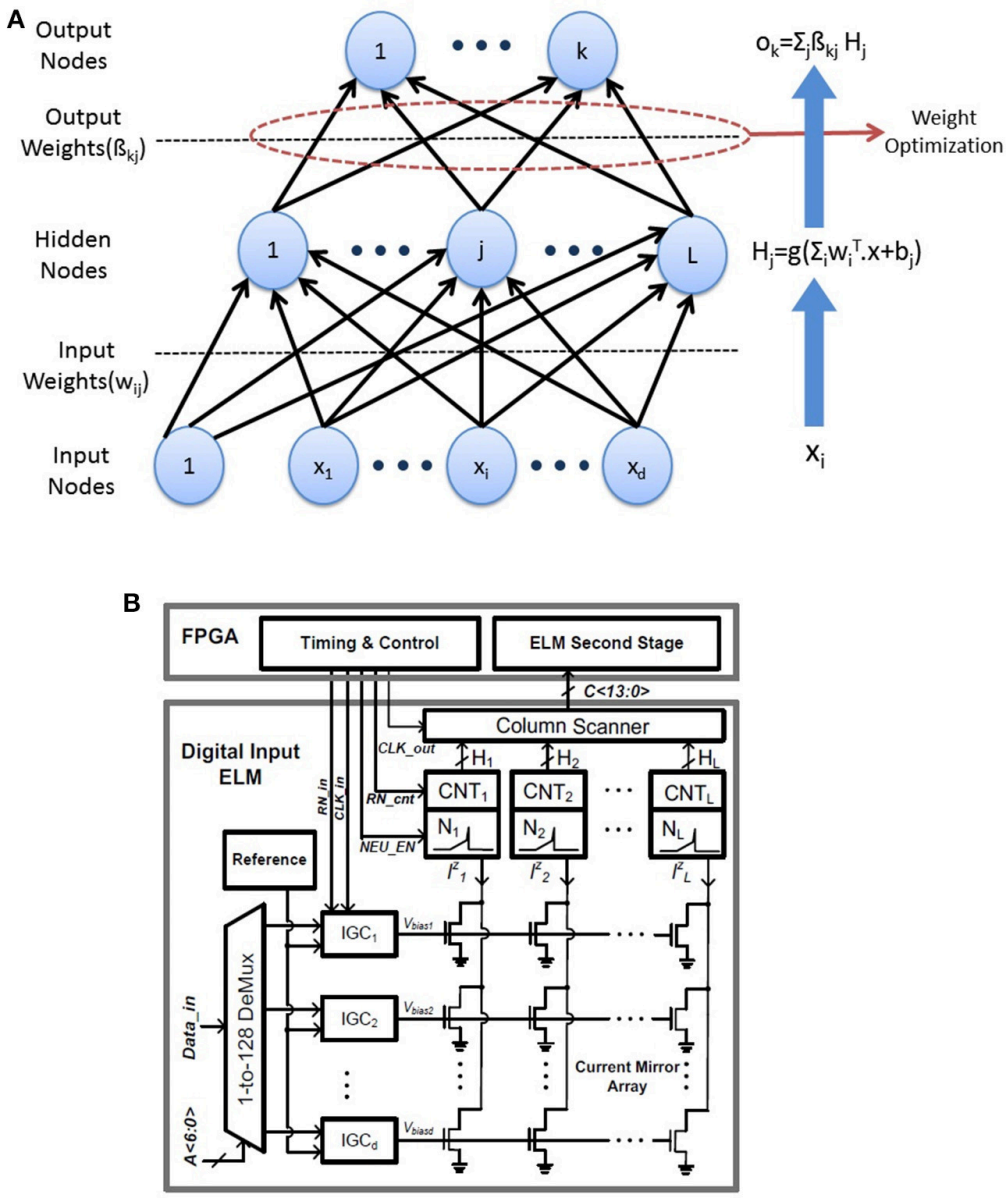
**(A)** ELM network architecture: The weights *w*_*ij*_ in the first layer are random and fixed while only the second layer weights need to be trained. **(B)** Architecture of the neuromorphic ELM IC (adapted from Patil et al., 2015).

The ELM is a three layer feedforward neural network introduced in Huang et al. (2006) shown in Figure 5A. The output of the ELM network with L hidden neurons is given by:

(2.3.1) $$o=\sum_{i}^{L}\beta_{i}H_{i}=\sum_{i}^{L}\beta_{i}g(w_{i}^{T}x+b_{i})$$

where *x* is a *d*-dimensional input vector, *b*_*i*_ is the bias of individual neurons, *w*_*i*_ and β_*i*_ are input and output weights respectively. *g*(.) is the non-linear activation function (sigmoid function is commonly used) and *h*_*i*_ is the output of the *ith* hidden neuron. While the weights *w*_*i*_ and *b*_*i*_ are chosen from any random distribution and need not be tuned, the output weights β_*i*_ need to be tuned during training. So the basic task in this architecture is to find the least square solution of β given targets of training data:

(2.3.2) $$Minimize_{\beta }:\;{\left\|H\beta -T\right\|}^{2},$$

where T is the target of training data. The optimal solution of β is given by

(2.3.3) $$\tilde{\beta }=H^{†T},$$

where *H*^†^ is the Moore-Penrose pseudoinverse of H (Penrose, 1955). The simplest method to find *H*^†^ is using orthogonal projection:

(2.3.4) $$\begin{array}{l} H^{†}={\left(H^{T}H\right)}^{-1}H^{T}\;if\;H^{T}H\;is\;non-singular\\ H^{†}=H^{T}{\left(HH^{T}\right)}^{-1}\;if\;HH^{T}\;is\;non-singular. \end{array}$$

Moreover, taking advantage of the concepts from ridge regression (Hoerl and Kennard, 1970), a constant is added to the diagonal of *H*^*T*^*H* or *HH*^*T*^ which results in a solution that is more stable and has better generalization performance. *C* is a tunable hyperparameter. Several regularization techniques have been explored for determining the optimal value of *H* to reduce training time and number of hidden neurons (Huang et al., 2012). The simple architecture of the ELM network makes it a suitable candidate for hardware implementation.

#### 2.3.2. Extreme learning machine: hardware

For the classification task, we have used software ELM as well as hardware measurements on the neuromorphic ELM chip described in Yao and Basu (2017).

The digital implementations of ELM can benefit from the software simulations of the ELM shown in this paper. The architecture of the ELM chip is shown in Figure 5B. The 128 input digital values are converted to analog currents using current mode DACs which are multiplied by random weights in a 128 × 128 current mirror array (CMA). The random weights are generated by the physical mismatch of transistors in the CMA. The 128 output currents are converted to spikes using an array of 128 integrate and fire neurons. The corresponding firing rates are obtained by an array of digital counters while the second stage of ELM is performed in digital on a FPGA. While the software ELM uses random weights with a uniform random distribution, the chip generates random weights *w*_*ij*_ with lognormal distribution. This is due to the exponential relation of current and threshold voltage (*V*_*T*_) in the sub-threshold regime which leads to mismatch induced weights of the form

(2.3.5) $$\begin{array}{r} w=e^{\Delta V_{T}/U_{T}} \end{array}$$

where Δ*V*_*T*_ denotes mismatch between threshold voltages of a pair transistors forming a current mirror. However, lognormal distributions have positive mean and software simulations show that zero mean weights result in higher classification accuracy. Hence, a simple digital post-processing is used on the outputs to obtain zero mean random numbers. Instead of directly feeding the chip output *h*_*i*_ to the second stage, the difference $h_{i}^{\prime }$ of neighboring neurons were used. So, the modified output of the hidden layer is given by:

(2.3.6) $$\begin{array}{r} h_{i}^{\prime }=h_{i}-h_{\left(i+1\right)mod\left(128\right)},i=1,2,..,128 \end{array}$$

As shown in Patil et al. (2015), any weight distribution *w*_*ij*_ can become a zero mean distribution $w_{ij}^{\prime }$ using this technique. We will refer to this as log difference weight for the rest of this paper. Finally, instead of using typical non-linearities like sigmoid or tanh as *g*(.), we have used an absolute value (abs) function as the preferred non-linearity. While software simulations show similar or slightly better classification accuracy for an absolute value non-linearity compared to typical non-linearities, it has several other advantages over them. Absolute value is a non-saturating non-linearity and so feature vectors need not be normalized before being passed to the ELM unlike saturating non-linearities.This reduces the computational burden. Moreover, the hardware implementation of abs non-linearity is much simpler than sigmoid or similar non-linearities.

## 3. Hardware complexity

In this section we will discuss the hardware complexity comprising computations and memory requirements for the classifier and the two feature extraction methods described earlier. For our calculation, we assume that the time stamp of a spike is encoded using 32 bits and the channel address of the spike is 6 bits. The average number of spikes per sample is assumed to be *N*_*sample*_ and the spike counter size is *b*_*counter*_ bits. The number of computations (*N*_*comp*_) can be written as the sum of two components:

(3.0.1) $$\begin{array}{r} N_{comp}=N_{feature}+N_{ELM} \end{array}$$

where *N*_*feature*_ is the number of computations for feature extraction while *N*_*ELM*_ is the number of computations required for classification by ELM.

The total memory required (*M*_*total*_) can be written as sum of two components:

(3.0.2) $$\begin{array}{r} M_{total}=M_{feature}+M_{ELM} \end{array}$$

where *M*_*feature*_ is the memory required for feature extraction while *M*_*ELM*_ is the memory required for classification by ELM.

### 3.1. Feature extraction

#### 3.1.1. Fixed number of bins

For the fixed number of bins method, the entire sample needs to be stored first and bin sizes are to be determined later. So, the memory required to store the spike information of an entire sample (time stamp and channel count) is

(3.1.1) $$\begin{array}{r} M_{samples}=38\times N_{sample}\;bits \end{array}$$

Now, if the number of bins is *B*_*sta*_, a total of *B*_*sta*_ × *C* counters are required to count the spikes and produce the feature vector. Therefore, the memory required to store a feature vector is given by:

(3.1.2) $$\begin{array}{r} M_{feature\text{\_}vector}=B_{sta}\times C\times b_{count}\;bits \end{array}$$

So, from Equations 3.1.1 and 3.1.2 the total memory requirement for fixed number of bins method is

(3.1.3) $$\begin{array}{rcl} M_{feature} & = & 38\times N_{sample}+B_{sta}\times C\times b_{count}\;bits\\ = & 38\times B_{sta}\times C\times {\bar{n}}_{spikes}+B_{sta}\times C\times b_{count}\;bits \end{array}$$

In terms of computations, there will be a counter increment for each spike resulting in *N*_*sample*_ operations per sample. Also, for each spike, the time stamp needs to be compared with the bin boundary to determine when to reset counters. Hence the total number of operations per sample is given by:

(3.1.4) $$\begin{array}{r} N_{feature}=N_{sample}+N_{sample}=2N_{sample} \end{array}$$

#### 3.1.2. Fixed bin size

For the fixed bin size method, the feature vectors are produced directly from the sample as the bin sizes are pre-determined. Thus, there is no need for storing the sample in memory. The only memory required in fixed bin size method is for storing the feature vectors. Since we cascade 10 bin outputs to produce a feature vector in this method, using calculations similar to above, we get:

(3.1.5) $$\begin{array}{r} M_{feature}=M_{feature\text{\_}vector}=10\times C\times b_{count}\;bits \end{array}$$

Finally, the total number of operations per sample is the total number of counter increments which is equal to the number of spikes used to produce the feature vector. So,

(3.1.6) $$\begin{array}{r} N_{feature}=N_{used}=min\left\{10\times C\times {\bar{n}}_{spikes},B_{fix}\times C\times {\bar{n}}_{spikes}\right\}, \end{array}$$

For the fixed bin size method, the memory requirement is significantly less than the fixed number of bins method as there is no need for storing the entire sample before feature extraction. Furthermore, pre-determined bin sizes enable this method to be compatible with real-time speech recognition systems. The significant advantage of this method over the fixed number of bins method in terms of memory and energy requirements is further quantified in section 4.3.

### 3.2. Classification

*N*_*ELM*_ again has two parts due to multiply and accumulate (MAC) in the first and second layers of the network. Hence, *N*_*ELM*_ is given by the following:

(3.2.1) $$\begin{array}{r} N_{ELM}=D\times L+L\times C_{o} \end{array}$$

where *C*_*o*_ is the number of output classes, *D* is the dimension of the feature vector and *L* is the number of hidden nodes. For our classification problem, number of output classes *C*_*o*_ = 11. Moreover, calculating log difference weights requires some additional subtractions (= *L*). Hence, the final value of *N*_*ELM*_ is given by:

(3.2.2) $$\begin{array}{r} N_{ELM}=D\times L+L\times C_{o}+L \end{array}$$

Finally, the amount of memory (*M*_*ELM*_) needed by the classifier is given by:

(3.2.3) $$\begin{array}{r} M_{ELM}=D\times L\times b_{W}+L\times C_{o}\times b_{\beta } \end{array}$$

where *b*_*W*_ and *b*_β_ denote the number of bits to represent the first and second layer weights.

The energy requirement for the ELM in the custom implementation will depend on the energy required for each of these operations. Since multiplications are dominant, *E*_*MAC*_ is the prime concern. Since it has been shown that $E_{MAC}^{ana}$ < $E_{MAC}^{dig}$ for the first stage with maximum number of multiplies (Chen et al., 2016), we have used an analog neuromorphic ELM hardware in this work. However, the findings of this work are applicable to a digital implementation of ELM on ASIC or on a microprocessor.

## 4. Results

### 4.1. Software simulations

In this section, we show the classification accuracies for different pre-processing strategies described in section 2.2 using a software ELM with uniform random weights and log difference weights. Though there are 64 (max. channel count) channels available in AEREAR2, only the first 54 channels were active for all the samples, therefore C = 54. All the results were obtained by averaging the classification accuracies over five randomized 90–10% train-test splits.

#### 4.1.1. Fixed number of bins (1B, 2B)

**Figure 6:**
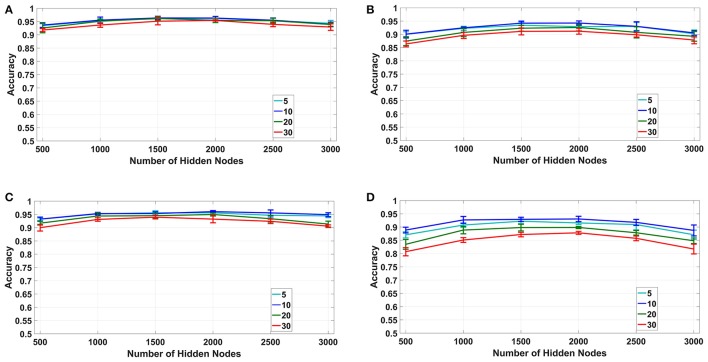
Fixed number of bins: Accuracy vs. number of hidden nodes for different number of bins. **(A,B)**: Time based binning (1B):10 bins per sample shows highest overall accuracy. **(C,D)**: Spike count based binning (2B): 10 bins per sample shows highest overall accuracy.

For the fixed number of bins method, we have used *B*_*sta*_ = 5, 10, 20, and 30 bins per sample for both time based and spike count based modes with number of hidden nodes in the classifier varying from *L* = 500 to 3, 000. The results for this experiment are plotted for both uniform random and log difference weights in Figures 6A,B for time based and in Figures 6C,D for spike based binning respectively. It can be seen that, for both modes, *B*_*sta*_ = 10 bins per sample produced maximum overall classification accuracy of around 96% for uniform random and 93.5% for log difference weights respectively. Also, the accuracies tend to initially increase with increasing values of *L* but eventually saturate and start decreasing due to over-fitting.

#### 4.1.2. Fixed bin size (1A, 2A)

**Figure 7:**
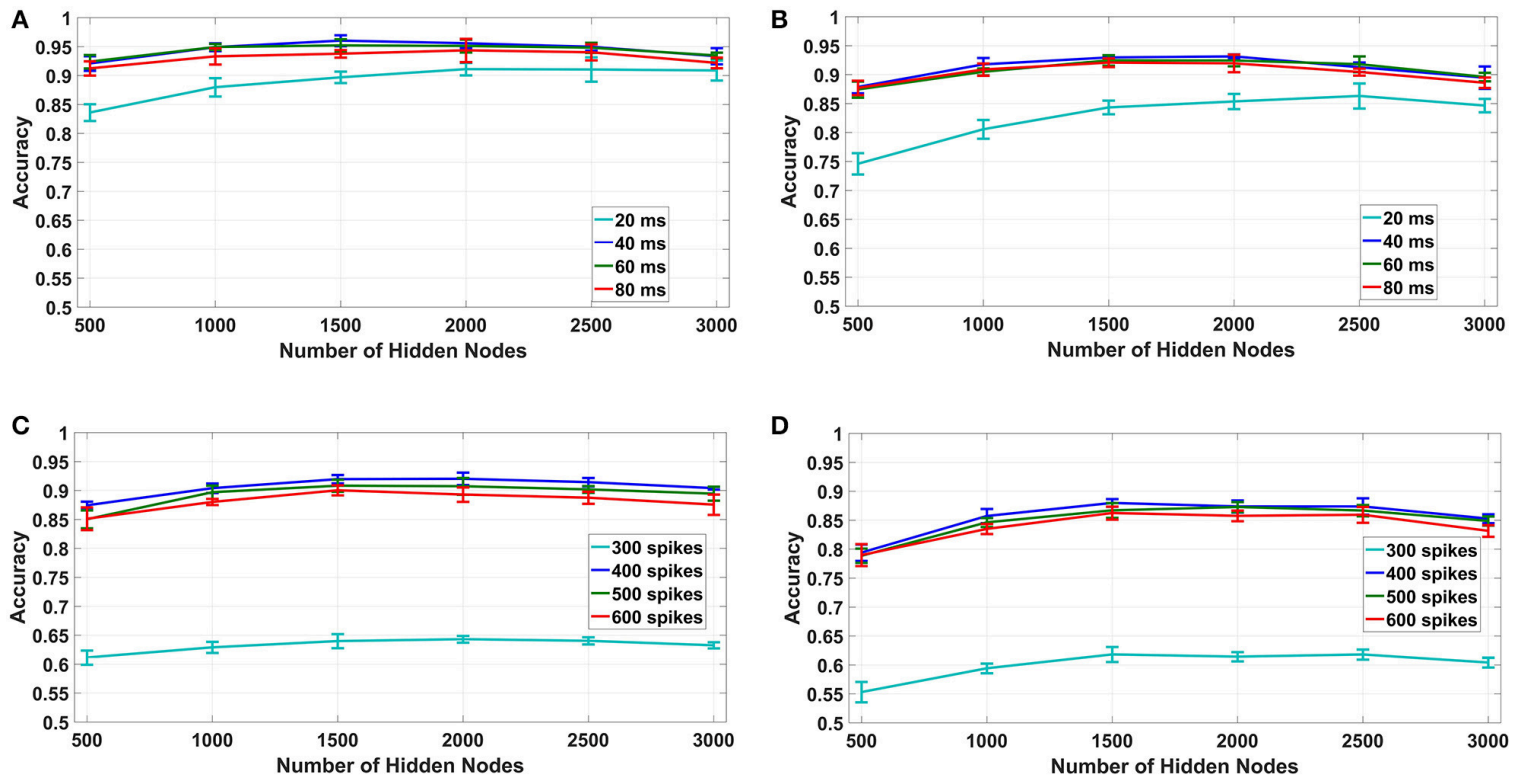
Fixed bin size: accuracy vs. number of hidden nodes for different bin sizes. **(A,B)**: Time based binning (1A): 40 ms bin size shows highest overall accuracy. **(C,D)**: Spike count based binning (2A): 400 spikes/bin shows highest overall accuracy.

For the fixed bin size method (1A, 2A in Table 1), we have used 10–40ms bin sizes for time based binning and 300 spikes/bin to 600 spikes/bin bin sizes for spike count based binning with number of hidden nodes varying from 500 to 3, 000.The results for this experiment are plotted for both uniform random and log difference weights in Figures 7A,B for time based and in Figures 7C,D for spike based binning respectively. It can be seen that, for time based mode, the maximum overall classification accuracy was obtained for 40 ms. We tried a bin size of up to 80 ms and found that the accuracy decreases beyond 40 ms. This is probably due to the fact that, while larger bin sizes ensure less loss of information at the end of a digit, it produces very small number of bins for shorter samples which results in their misclassification. For spike count based mode the maximum overall classification accuracy was obtained for 400 spikes/bin. Interestingly, even with fixed bin size features, we can obtain classification accuracies ~95% for time based binning in both cases of uniform and log difference weights. Hence, this points to a method for low hardware complexity feature extraction that also allows usage of analog sub-threshold ELM circuits with log difference weights. Second, the trend of increasing accuracies with increasing temporal bin size is due to the ELM being able to access larger parts of the speech sample. Lastly, the difference between spike count based binning and time based binning is very large in this case indicating that spike count alone is not a good distinguishing feature for fixed bin size.

#### 4.1.3. Combined binning

**Figure 8:**
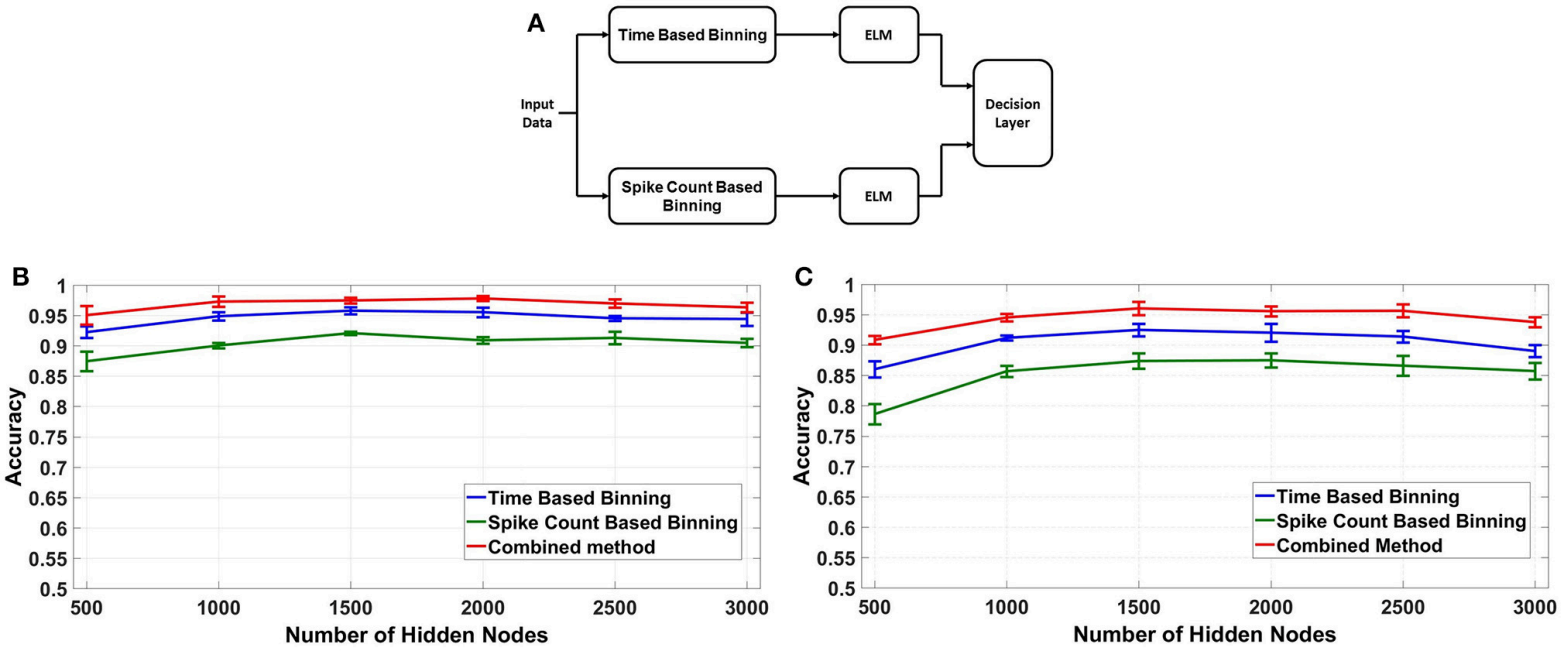
**(A)** Combined Binning architecture for fixed bin size case by fusing the decisions of two ELMs operating in time based and spike count based modes respectively. **(B,C)** Comparison of binning modes, fixed bin size: Accuracy vs. Number of Hidden Nodes using different binning modes for fixed bin size, Combined Mode shows highest overall accuracy, comparable to fixed number of bins.

Out of the two binning strategies described in this paper, the fixed bin size method is more convenient to implement from a hardware perspective. Moreover, the memory and energy requirements of the fixed bin size method are much less than its counterpart as discussed in section 4.3. But as we have shown in section 4.1.2, the best case accuracy of the fixed bin size method is typically 2–3% less than that of fixed number of bins method. This is due to two factors: lack of input temporal normalization and loss of information due to discarded bins. To increase the accuracy of the fixed bin size method, we adopted a combined binning approach as shown in Figure 8A. In this fixed bin size strategy, the input data is processed in parallel using both time based and spike count based binning.The feature vectors produced are applied to their respective ELMs and the ELM outputs are combined (added) in the decision layer. The final output class is defined as the strongest class based on both strategies. Figures 8B,C compares the best case accuracies of time based binning (40 ms bin size), spike count based binning (400 spikes/bin bin size) and combined binning mode (combination of both). The combined binning mode not only outperforms both the time and spike count based modes, but also shows accuracies similar to the best case accuracies of fixed number of bins method for both type of weights.The reasons for this increased accuracy is further discussed in section 5.

### 4.2. Hardware measurements

**Figure 9:**
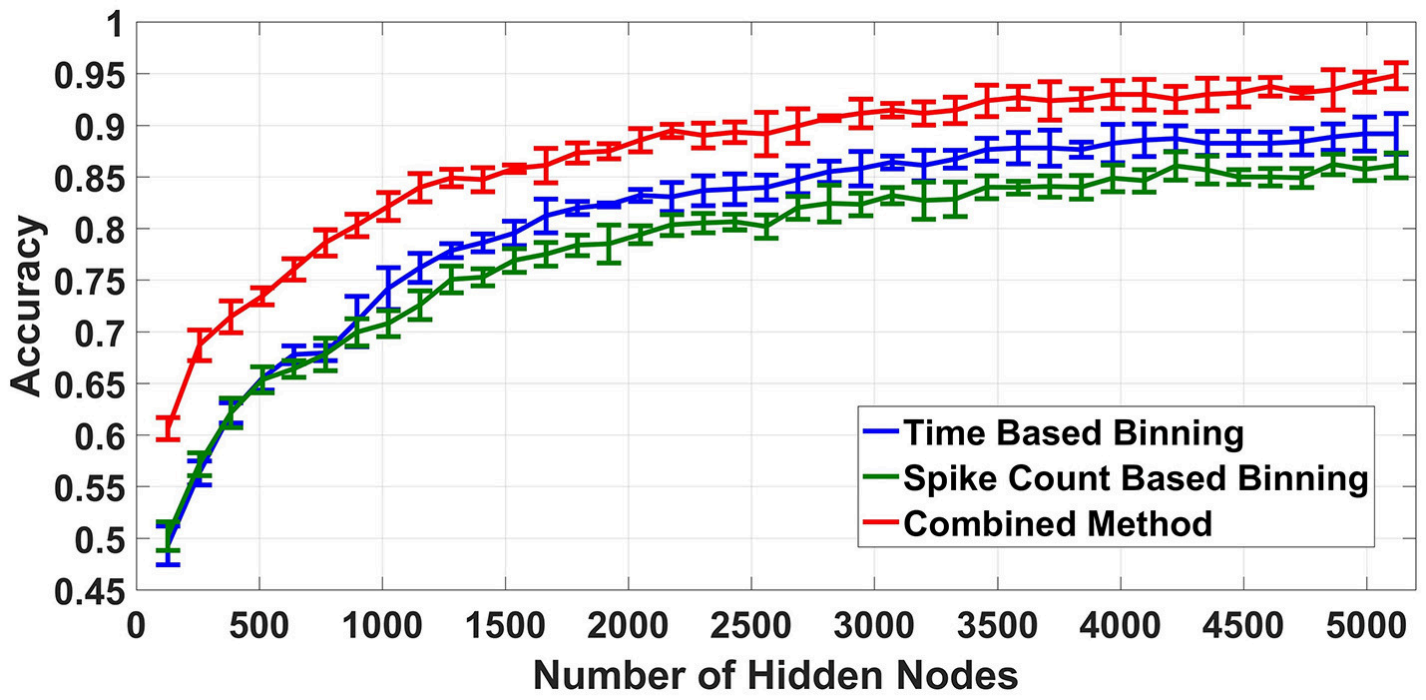
Hardware classification accuracies for different binning strategies, Combined Binning strategy shows highest classification accuracy.

Finally, the proposed feature extraction methods were tested on a neuromorphic ELM IC described in Yao and Basu (2017) by feeding the chip with feature vectors produced by the methods described above. Due to the long testing times needed, we only tested the best accuracy cases of time based binning (40 ms bin size), spike count based binning (400 spikes/bin bin size) and combined binning (combination of the two). The accuracies obtained are shown in Figure 9. The optimum accuracy obtained by time based binning is slightly higher than that of spike count based binning while combined binning approach outperforms both of the methods. However, comparing this result with the earlier software simulations, we notice two differences. First, the accuracies obtained are slightly less than software and second, the accuracy increases with increasing *L*.

Possible reasons for this reduction in accuracy and its subsequent increase with increasing *L* are discussed in section 5.

### 4.3. Memory and energy requirement (highest accuracy case is marked red)

In this section, we will determine the memory and energy requirements of different post processing methods described. We have used the formulae derived in section 3 to determine the memory requirement and the computational complexity of different strategies. Moreover, we used the specifications of Apollo2 Ultra-Low Power Microcontroller for calculating pre-processing energy requirement (10μ*A*/*MHz* at 3.3*V*^1^) and specifications of the neuromorphic ELM chip for calculating the classification energy requirement (0.47*pJ*/*MAC*, Yao and Basu, 2017). Tables 2, 3 show the memory requirement, computational complexity and average energy per sample of fixed number of bins and fixed bin size strategies assuming 1500 hidden nodes for the ELM. If we compare the best accuracy cases of both fixed bin size and fixed number of bins methods, these results show that fixed binning requires ~50× less memory for feature extraction (~3× overall) and ~30% less energy compared to that of fixed number of bins method. Furthermore, as the combined binning requires approximately twice the memory and computational complexity than that of the simple time or spike count based binning methods, we can conclude that the combined binning strategy is able to produce accuracies similar to fixed number of bins method using ~25× less memory for feature extraction (~1.5× overall). Moreover, since the neuromorphic ELM chip uses mismatch induced random weights for the first layer of the ELM, no memory is required to store the first layer weights. Only, the second layer trained weights need to be stored in memory. The minimum resolution of the second layer weights (*b*_β_) required for no loss of accuracy is found to be 8 bits.

**Table 2:** Memory and energy requirements for fixed number of bins method (1B,2B).

Bins/ Sample	5	10	20	30
Memory Required (Feature Extraction) (Kbits)	213	215	219	223
Memory Required (ELM Layer 2) (Kbits)	132	132	132	132
No.of Ops/sample (Feature Extraction) (Kops)	11	11	11	11
No. of MACs/sample (ELM Layer 1) (KMACs)	405	810	1,620	2,430
No. of MACs/sample (ELM Layer 2) (KMACs)	18	18	18	18
Energy Required (nJ/sample)	3,061	3,251	3,632	4,013

**Table 3:** Memory and energy requirements for fixed bin size method (1A, 2A). Highest accuracy cases are marked red.

	**Time based binning**				**Spike count based binning**			
Bin Size	10 ms	20 ms	30 ms	40 ms	300 spikes /bin	400 spikes /bin	500 spikes /bin	600 spikes /bin
Memory Required(Feature Extraction) (Kbits)	4.3	4.3	4.3	4.3	4.3	4.3	4.3	4.3
Memory Required(ELM Layer 2) (Kbits)	132	132	132	132	132	132	132	132
No.of Ops/sample (Feature Extraction) (Kops)	0.7	1.4	1.8	2	2	3	4	5
No. of MACs/sample (ELM Layer 1) (KMACs)	810	810	810	810	810	810	810	810
No. of MACs/sample (ELM Layer 2) (KMACs)	18	18	18	18	18	18	18	18
Energy Required (nJ/sample)	2,232	2,301	2,340	2,360	2,360	2,459	2,558	2,657

## 5. Discussion

### 5.1. Hardware vs. software ELM

**Figure 10:**
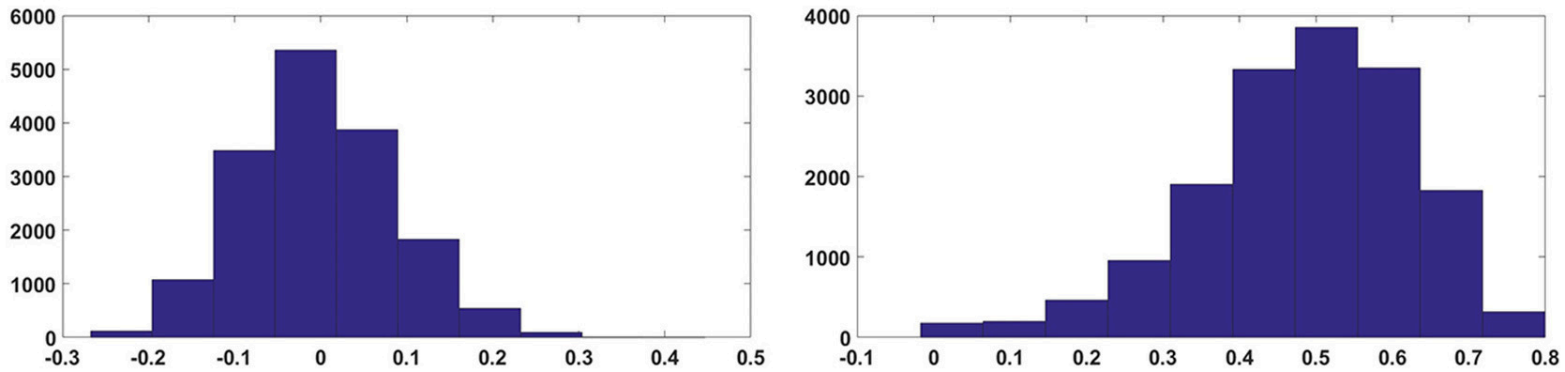
Histogram of correlation coefficients of input weights.

One key observation from the results obtained is that the hardware ELM requires larger number of hidden nodes to obtain accuracies similar to the software simulations (compare Figure 8 and Figure 9). While software simulations required around 2,000 hidden nodes to obtain optimum accuracy, the hardware required more than 5,000 hidden nodes to obtain comparable accuracies.This discrepancy can be ascribed to the higher correlation between input weights in the ELM IC. In an ideal ELM, the input weights of are assumed to be random and so, the correlation between successive columns of weights should be low. But in the ELM IC, the correlation between successive columns of weights are relatively higher due to chip architecture. Since the DACs converting the input digital number to a current is shared for each row, mismatch between the DACs introduce a systematic mismatch between rows. This systematic variation of the input weight matrix results in increased correlation between columns of input weights. Figure 10 shows the histogram of inter column correlation coefficients for hardware weights and software simulated log normal weights. Greater correlation between hardware weights can alternatively thought of as a reduction in effective number of uncorrelated weights and thereby, a reduction in number of uncorrelated hidden nodes compared to software simulations. Therefore, the “effective” number of hidden nodes in hardware case is in fact smaller than the number of hidden nodes used in the IC. This explains the requirement of higher number of hidden nodes in hardware to match the performance of software simulations.

**Figure 11:**
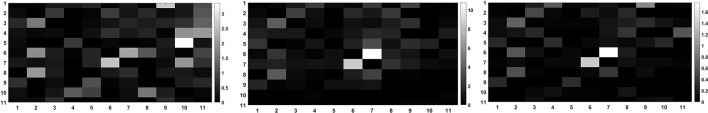
Confusion matrices for different binning strategies exhibit peaks at different locations for time based and spike count based binning. Hence, a combination of these two methods can eliminate some of these errors.

Another significant observation about the experimental results is that the combined strategy consistently outperforms both time based binning and spike count based binning methods for software as well as hardware simulations. This can be attributed to the synergy produced by combining two disparate representations of the input data (time based features and spike count based features) using a decision layer. To prove the importance of using two different representations, we have obtained the average confusion matrices for both time based binning and spike count based binning using several randomized training and testing sets. The resulting confusion matrices are plotted alongside the confusion matrix for the combined strategy in Figure 11. It can be clearly seen from the confusion matrices that while some of the peaks of the confusion matrices are at the same locations for both time based and spike count based methods, a significant number of minor peaks are at different locations. Therefore, a significant number of those misclassifications occurring for only one of the two binning methods are correctly classified in the combined strategy. This claim has been further quantitatively analyzed in Appendix.

### 5.2. Comparison with other methods

Next, we compare our results with reported accuracies in existing literature using the N-TIDIGITS18 dataset. For fixed bin size strategy, Neil and Liu (2016) obtained an accuracy of 87.65% using CNN and an accuracy of 82.82% using GRU RNN. Anumula et al. (2018) also obtained 88.6% accuracy using GRU RNN and 86.1% accuracy using LSTM RNN for the same feature extraction technique. For fixed number of bins strategy, Abdollahi and Liu (2011) obtained an accuracy of 95.08% using SVM. Thus, we can see that the accuracies reported in this paper outperform those obtained using fixed bin size or fixed number of bins techniques in existing literature. The best case accuracies obtained in this paper are comparable to that of MFCC based features in previous works [using MFCC based features, (Abdollahi and Liu, 2011) obtained an accuracy of 96.83% using SVM while (Anumula et al., 2018) obtained an accuracy of 97.90% using GRU RNN]. However, this comparison is imperfect since we need to account for the power needed in generating mode complex features like MFCC. Tsai et al. (2017) has shown that the power required for MFCC feature extraction is 122 mW on FPGA based implementation and 62.3 mW on ARM based implementation for TIDIGITS dataset using a 32 ms frame size. This is significantly higher than that of feature extraction techniques described in this paper (Tables 2, 3). Also, it is difficult to compare power dissipation of RNN approaches since very few hardware implementations of these networks are reported. As one example, Gao et al. (2018) reports a Delta RNN network that uses ≈453*K* operations per frame of 25 ms (excluding FFT operations to generate features) which is quite comparable to the number of operations needed by the ELM first stage. However, it should be noted that the ELM first stage operations were simple random multiplications which could be easily implemented in low pwoer using analog techniques while the same cannot be said for the RNN.

### 5.3. Real-time detection of word occurrence

For the classification of the dataset so far we have assumed that the start and end of a digit is clearly marked for both training and testing data. But for real time applications, this assumption will not hold. So, we have decided to employ a sliding window technique for automatic detection of start and end of a digit. For the spike N-TIDIGITS18 dataset we have used, no noise was added to the waveforms of the original TIDIGITS dataset. So, the detection of start and end of the digit will become a relatively trivial task. However, the more challenging task is to detect the start and end of the signal in presence of noise. Therefore, we have implemented a threshold-based start and end detection using a sliding window assuming presence of noise. The algorithm detects the start of a digit if the total spike count within the window is higher than the given threshold and rejects the frame as noise if the total spike count is less than the threshold. Once the start of a digit is detected, the upcoming spikes are assumed to be part of the digit until the total spike count within a window is less than the threshold for a certain number of consecutive windows. At this point, the last window where the spike count was higher than the threshold is assumed to be the end of the digit. This ensures that the false end detection is avoided in case there are low spike count windows within the digit. We have set the threshold as a certain % of average spike count per window over all samples and the number of consecutive low spike count windows required to determine the end of a digit is a parameter dependent on the sliding window size.

We have tested this algorithm on best accuracy cases of both fixed number of bins strategy (time based binning, 10 bins/sample) and fixed bin size strategy (time based binning, bin size = 40 ms). We used a non-overlapping sliding window size of 40 ms and 2 consecutive windows with sub-threshold spike count for end detection. For fixed bin size strategy, the accuracy remained same for 10% threshold level and decreased by 0.8% for 20% threshold level. For fixed number of bins strategy, the reductions in accuracy were 2.5% and 3.6% respectively for 10% and 20% threshold level respectively. The diminished effect of start and end detection on the classification accuracy for fixed bin size strategy can be attributed to its indifference toward digit duration and thereby exact start and end time unlike its counterpart. Thus, the fixed bin size strategy seems relatively more noise robust.

In this proposed algorithm, the loss of accuracy stems from three sources, (a) loss of bins at the beginning, (b) loss of bins at the end and (c) loss of part of the digits due to false detection. For the fixed bin size case, only (c) is the major contributor to loss in accuracy while for fixed bin size case, all three factors contribute to the accuracy loss. Moreover, this sliding window technique introduces some additional latency depending upon the number of sub-threshold spike count windows used for end detection.

## 6. Conclusion

In this paper, we have presented several low-complexity feature extraction techniques to construct an end-to-end speech recognition system using a neuromorphic spiking cochlea and neuromorphic ELM IC. Moreover, the computational complexity, power requirement and memory requirement of the proposed techniques were calculated. Furthermore, we have used both software and hardware simulations of the neuromorphic ELM IC to obtain high classification accuracies (~96%) for the N-TIDIGITS18 dataset.

The proposed fixed number of bins and fixed bin size methods presented a clear trade-off between classification accuracy and hardware overhead where using fixed number of bins gives ~2-3 % higher accuracy with ~3× more hardware overhead compared to the fixed bin size method. Our strategy of combining two different feature space representations of the input data gives high classification accuracy while using ~ 25× less memory compared to the fixed number of bins method. So far, the feature extraction block of our proposed architecture is simulated in software only. In future, we plan to implement the feature extraction block using a microcontroller to produce a fully hardware based neuromorphic speech recognition system based on the low-power component prototypes Yang et al. (2016). Moreover, we plan to use our proposed architecture for other speech and audio recognition problems including speaker identification.

## Author contributions

All the authors have contributed in varying degrees to different aspects of this paper. JA contributed in data analysis, software simulations, drafting and revising the manuscript. AP has contributed in experiment design and data collection using ELM IC. XL has contributed in experiment design and data acquisition using silicon cochlea. YC has contributed in data analysis and hardware data collection using ELM IC. S-CL has contributed in overall conception and design of the experiments and revising the manuscript. AB has also contributed in conception and design of the experiments and drafting and revising the manuscript.

### Conflict of interest statement

The authors declare that the research was conducted in the absence of any commercial or financial relationships that could be construed as a potential conflict of interest.

## Figures and Tables

**Figure A1:**
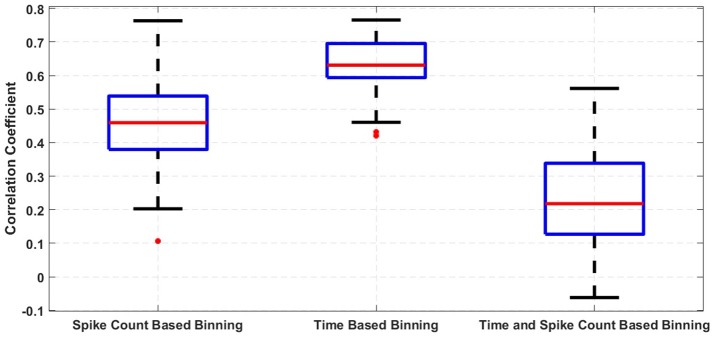
Correlation between confusion matrices.

## Footnotes

^1^http://ambiqmicro.com/apollo-ultra-low-power-mcu/apollo2-mcu/

## Appendix

### Further discussion on confusion matrices

To quantitatively analyze our hypothesis in section 5 that the correlation matrices produced by time based binning and spike count based binning have peaks at different locations, we have used correlation coefficients. We have calculated the correlation coefficients between confusion matrices produced by time (and spike count) based binning for different randomizedtraining and testing sets. We have also obtained the cross-correlation coefficients between confusion matrices produced by time and spike count based binning for same training and testing sets. The spread of the correlation coefficients obtained is shown using the box-plots in Figure A1. It is quite evident from the box-plots that confusion matrices produced by the same feature extraction method for different training and testing sets are highly correlated while confusion matrices produced by different feature extraction methods for same training and testing set have lower correlation.
